# Genomic organization of repetitive DNAs highlights chromosomal evolution in the genus *Clarias* (Clariidae, Siluriformes)

**DOI:** 10.1186/s13039-016-0215-2

**Published:** 2016-01-20

**Authors:** Nuntiya Maneechot, Cassia Fernanda Yano, Luiz Antonio Carlos Bertollo, Nuntaporn Getlekha, Wagner Franco Molina, Sukhonthip Ditcharoen, Bundit Tengjaroenkul, Weerayuth Supiwong, Alongklod Tanomtong, Marcelo de Bello Cioffi

**Affiliations:** Department of Biology, Faculty of Science, Khon Kaen University, Muang District Khon Kaen, Thailand; Departamento de Genética e Evolução, Universidade Federal de São Carlos, São Carlos, São Paulo Brazil; Departamento de Biologia Celular e Genética, Centro de Biociências, Universidade Federal do Rio Grande do Norte, Natal, RN Brazil; Department of Veterinary Clinical Medicine, Faculty of Veterinary Medicine, Muang, Khon Kaen 40002 Thailand; Faculty of Applied Science and Engineering, Khon Kaen University, Nong Khai Campus, Muang, Nong Khai 43000 Thailand; Toxic Substances in Livestock and Aquatic Animals Research Group, Khon Kaen University, Muang, Khon Kaen 40002 Thailand

**Keywords:** Chromosomal rearrangements, FISH, Karyotype evolution, Molecular cytogenetics, Centric fission

## Abstract

**Background:**

The genus *Clarias* (Clariidae, Siluriformes) contains at least 61 species naturally spread over vast regions of Asia, India and Africa. However, *Clarias* species have also been introduced in many different countries and represent the most widespread catfishes in the world. These fishes are also known as “walking catfishes” due to their ability to move over land. A large degree of chromosomal variation has been previously found in this family, mainly using conventional cytogenetic investigations, with diploid chromosome numbers ranging between 48 and 100. In this study, we analyzed the karyotype structure and distribution of four repetitive DNA sequences (5S and 18S rDNAs and (CA)_15_ and (GA)_15_ microsatellites) in three *Clarias* species (*C. batrachus, C. gariepinus, C. macrocephalus*), as well as in a probable natural hybrid of the two latter species from different Thailand river basins.

**Results:**

*Clarias gariepinus* and *C. macrocephalus* had 2n = 56 and 2n = 54, respectively, as well as karyotypes composed mainly by metacentric and submetacentric chromosomes. Their karyotypes differed in the number and location of 5S and 18S rDNA sites and in the degree of microsatellite accumulation. An intermediate chromosomal pattern incorporating those of the parental species was found in the probable hybrid, confirming its interspecific origin. *Clarias batrachus* had 2n = 104 chromosomes and its karyotype was dominated by mainly acrocentric elements, indicating that unusual multiple centric fissions were involved in its karyotype differentiation. The karyotype of this species presented an unexpected dispersion of ribosomal DNAs, possessing 54 and 12 sites of 5S and 18S rDNAs, respectively, as well as a high accumulation and differential distribution of both microsatellite repeats, representing ‘hot spots’ for chromosomal rearrangement.

**Conclusion:**

Both conventional and molecular cytogenetic markers were useful tools for demonstrating remarkable evolutionary dynamism and highlighting multiple chromosomal rearrangements and hybridization events correlated with the notable karyotypic diversity of these walking catfishes.

## Background

The catfish family Clariidae comprises 14 genera and more than 115 species found in India, Syria, Southern Turkey, Southeast Asia and Africa, with the highest species diversity found in the latter [1]. The genus *Clarias* Scopoli, 1763, comprises at least 57 species and is widely distributed across Africa and Southeast Asia [2, 3]. These fishes are known as “walking catfishes” because they have an accessory air-breathing organ that allow them to survive for months in oxygen-poor water or even completely out of water [4]. Moreover, some species are able to migrate over land using wriggling movements. These characteristics and behaviour may affect the dispersal, speciation and genetic parameters of the population, potentially altering the processes from those commonly recognized in other obligatory freshwater fishes. In *Clarias* species examined to date, diploid chromosome numbers range between 2n = 48 and 2n = 56, with the exception of *C. pachynema* (2n = 66) and one population of C. *batrachus* (2n = 100) (Table 1). However, all chromosomal data were obtained by conventional Giemsa-stained chromosomes and molecular cytogenetic studies are still virtually absent.

**Table 1:** Review of available data on 2n, karyotypes and sex systems in the genus *Clarias*

Species	2n	NF	Karyotype	Sex system	Locality	Reference
*C. albopunctatus*	48	75	4 m + 23sm + 21a	ZW	Nigeria	[19]
*C. albopunctatus*	48	74	4 m + 22sm + 22a	ZZ	Nigeria	[19]
*C. anguillaris*	56	91	8 m + 27sm + 21a	ZW	Nigeria	[19]
*C. anguillaris*	56	90	8 m + 26sm + 22a	ZZ	Nigeria	[19]
*C. anguillaris*	48	–	27 m + 10sm + 3st + 8 t	–	Nigeria	[53]
*C. anguillaris*	56	–	33 m + 12sm + 2st + 9 t	–	Nigeria	[53]
*C. batrachus*	100	111	4 m + 7sm + 77a + 12mc	XY	Thailand	[54]
*C. batrachus*	56	–	–		China	[55]
*C. batrachus*	100	110	4 m + 6sm + 78a + 12mc	XX	Thailand	[54]
*C. batrachus*	50	88	16 m + 8sm + 14st + 12a		India	[56]
*C. batrachus*	50	96	18 m + 20sm + 8st + 4a		India	[57]
*C. batrachus*	54	–				[58]
*C. batrachus*	50	89	16 m + 11sm + 5st + 1at	ZW	India	[59]
*C. batrachus*	50	88	16 m + 10sm + 6st + 18a	ZZ	India	[59]
*C. batrachus*	51	89	16 m + 11sm + 5st + 18a + 1B-chromosome	ZW	India	[59]
*C. batrachus*	51	88	16 m + 10sm + 6st + 18a + 1B-chromosome	ZZ	India	[59]
*C. batrachus*	50	96	12 m + 18sm + 10st + 10 t	–	India	[60]
*C. batrachus*	50	90	11 m + 7sm + 2st + 34a	–	Malaysia	[61]
*C. batrachus*	54	74	12 m + 18sm + 10st + 14 t	–	India	[62]
C. batrachus	104	–	2 m + 4sm + 98st/a	-	Thailand	Present study
*C. camerunensis*	54	–	–	–	Africa	[63]
*C. camerunensis*	56	–	22 m + 20sm + 9st + 5 t	-	Nigeria	[53]
*C. ebriensis*	50	–	–	–	Africa	[63]
*C. ebriensis*	48	77	6 m + 23sm + 19a	ZW	Nigeria	[19]
*C. ebriensis*	48	76	6 m + 22sm + 20a	ZZ	Nigeria	[19]
*C. fuscus*	56	106	18 m + 24sm + 8st + 6a	XX	China	[54]
*C. fuscus*	56	106	19 m + 23sm + 8st + 6a	XY	China	[54]
*C. fuscus*	56	106	20 m + 22sm + 8st + 6a	XX	China	[64]
*C. fuscus*	56	106	20 m + 22sm + 8st + 6a	XY	China	[64]
*C. fuscus*	56	102	18 m + 14sm + 14st + 10a	XX,XY	China	[65]
*C. fuscus*	56	88	32 m/sm + 24st/a	–	Japan	[63]
*C. gariepinus*	56	89	8 m + 25sm + 23a	ZW	Africa, Israel	[37, 66, 67]
*C. gariepinus*	56	88	8 m + 24sm + 24a	ZZ	Africa, Israel	[37, 58, 67]
*C. gariepinus*	56	87	14 m + 17sm + 25a	ZW	Egypt	[60]
*C. gariepinus*	56	88	14 m + 18sm + 24a	ZZ	Egypt	[60]
*C. gariepinus*	56	102	20 m + 16sm + 10st + 10a	–	India	[60]
*C. gariepinus*	56	89	8 m + 25sm + 23a	ZW	Nigeria	[19]
*C. gariepinus*	56	88	8 m + 24sm + 24a	ZZ	Nigeria	[19]
*C. gariepinus*	56	96	21 m + 14sm + 5st + 16a	–	Malaysia	[61]
*C. gariepinus*	56	–	25 m + 14sm + 14st + 3 t	–	Nigeria	[53]
*C. gariepinus*	56	100	28 m + 6sm + 10a + 12 t	–	Turkey	[68]
*C. gariepinus*	56	100	24 m + 10sm + 10a + 12 t	–	Turkey	[68]
*C. gariepinus*	54	98	34 m + 10sm + 10 t	–	Nigeria	[69]
*C. gariepinus*	56	102	6 m + 12sm + 28st + 10a	–	Nigeria	[70]
*C. gariepinus*	56	98	30 m + 6sm + 6st + 14 t	–	Thailand	[71]
*C. gariepinus*	56	110	18 m + 20sm + 16st + 2a	–	Thailand	Present study
*C. jaensis*	54	–	22 m + 12sm + 5st + 15 t	–	Nigeria	[53]
*C. macrocephalus*	54	104	24 m + 20sm + 6st + 4a		Thailand	[54]
*C. macrocephalus*	54	98	22 m + 18sm + 4st + 10a	–	Malaysia	[61]
*C. macrocephalus*	54	104	22 m + 16sm + 12st + 4a	–	Thailand	Present study
*C. macromystax*	49	–	27 m + 10sm + 11st + 1 t	–	Nigeria	[53]
*C. platycephalus*	54	–	–		Africa	[63]
*C. pachynema*	66	–	30 m + 10sm + 16st + 10 t	–	Nigeria	[53]

The molecular organization and cytogenetic mapping of repetitive DNA elements, including satellites, multigene families and microsatellite repeats, have been analyzed in a large number of species and have demonstrated enormous potential for expanding our understanding of karyotype differentiation in fishes (reviewed in [5]). In fact, the correlation between the presence of high amounts of repetitive sequences and the higher number of chromosomal rearrangements has been widely demonstrated, since the accumulation of repetitive DNAs in particular genomic regions may prompt chromosome breakages, deletions, inversions and amplifications [6].

In this study, we analyzed the karyotype structure and distribution of four repetitive DNA sequences [5S and 18S rDNAs and (CA)_15_ and (GA)_15_ microsatellites] in three *Clarias* species (*C. gariepinus, C. macrocephalus* and *C. batrachus*) and in a probable natural hybrid of *C. gariepinus* and *C. macrocephalus* from different Thailand river basins with the aim of investigating their chromosomal differentiation and relationships. We observed remarkable chromosomal dynamism and karyotype characteristics that confirmed the hybridization of *C. gariepinus* x *C. macrocephalus*.

## Results

### Karyotypes

**Fig. 1:**
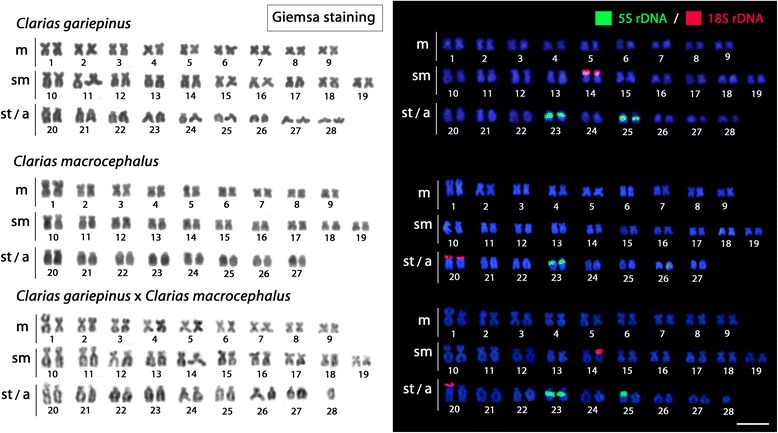
Karyotypes arranged from metaphase chromosomes of *Clarias gariepinus* (2n = 56), *Clarias macrocephalus* (2n = 54) and the natural hybrid of these species (2n = 55) after Giemsa staining and FISH with 18S rDNA (red) and 5S rDNA (green) probes. Note the intermediate level of distribution of the ribosomal sites in the hybrid specimen compared to the parental species. Scale bar = 5 μm

**Fig. 2:**
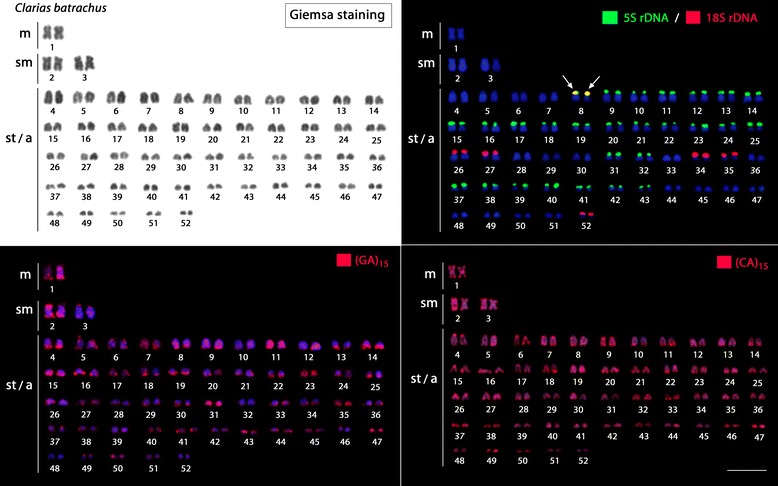
Karyotypes arranged from metaphase chromosomes of *Clarias batrachus* (2n = 104) after Giemsa staining and FISH with 18S rDNA (red), 5S rDNA (green), and (GA)_15_ and (CA)_15_ microsatellite probes. Note the high dispersion of 5S rDNA sites in the karyotype. Scale bar = 5 μm

No differences between male and female karyotypes were observed in any species. *Clarias gariepinus* showed a chromosome number of 2n = 56 (18 m + 20 sm + 18 st/a) and a fundamental number (NF) equal to 94. *Clarias macrocephalus* showed 2n = 54 (18 m + 20 sm + 16 st/a) and a NF of 92. The probable natural hybrid of *C. gariepinus* x *C. microcephalus* had a 2n between the two species (55) with a karyotype composed of 18 m + 20 sm + 17 st/a, and a NF equal to 91 (Fig. 1). *Clarias batrachus* showed a 2n = 104 (2 m + 4 sm + 98 st/a), with the NF equal to 110 (Fig. 2).

### Chromosome mapping of 5S and 18S rDNA sequences

The 18S rDNA probe hybridized to the subtelomeric/telomeric region of one medium-sized sm chromosomal pair in *C. gariepinus* and in one large st/a pair in *C. macrocephalus.* The 5S rDNA sequences were located in two small st/a pairs in *C. gariepinus* and in only one st/a pair in *C. macrocephalus*. Accordingly, the supposed hybrid of *C. gariepinus* x *C. microcephalus* had the intermediate number for both rDNA probes, with two 18S rDNA sites present in non-homologous chromosomes, in addition to three 5S rDNA sites (Fig. 1).

However, *C. batrachus* showed a surprising increase in the number of both rDNA classes, with six chromosomal pairs harbouring 18S rDNA sites and 27 chromosomal pairs harbouring 5S rDNA sites, including a syntenic condition in one pair (Fig. 2).

### Chromosome mapping of microsatellite sequences

**Fig. 3:**
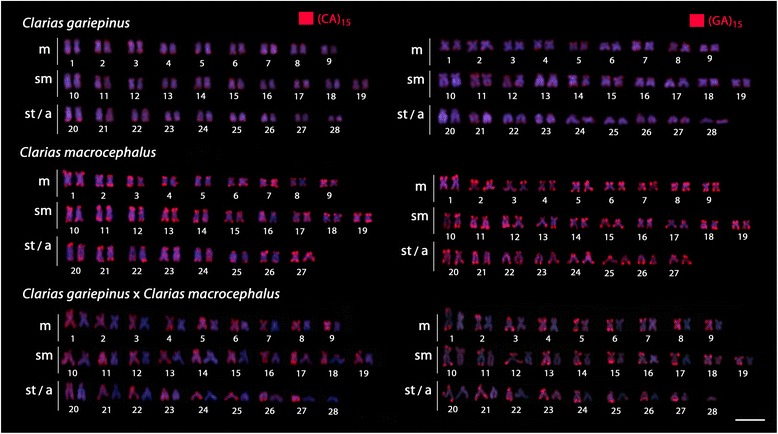
Karyotypes arranged from metaphase chromosomes of *Clarias gariepinus* (2n = 56), *Clarias macrocephalus* (2n = 54) and the natural hybrid of these species (2n = 55) after FISH with (GA)_15_ and (CA)_15_ microsatellite probes. Note the weak distribution of both microsatellites in *C. gariepinus*, their strong accumulation in *C. macrocephalus* and the intermediate distribution pattern in the hybrid specimen. Scale bar = 5 μm

In *C. gariepinus*, faint hybridization signals were found for these sequences across all chromosomes. However, in *C. macrocephalus* and *C. batrachus,* both (CA)_15_ and (GA)_15_ microsatellites were highly accumulated along all chromosomes. Significantly, the likely interspecific hybrid had the exact intermediate pattern of *C. gariepinus* and *C. macrocephalus*, with 27 chromosomes presenting a strong hybridization pattern, and the other 28 presenting weak accumulation of these sequences (Figs. 2 and 3).

## Discussion

### Karyotype variability among Clarias species

Clariidae is a very well defined monophyletic family based on the presence of a unique arborescent suprabranchial organ that enables the species to breathe atmospheric oxygen [4]. Nevertheless, Clariidae species are remarkable for the considerable variation in their external morphology [7–9]. Therefore, the chromosomal divergence among *Clarias* species (Table 1) also parallels the morphological differentiation of the clariid catfishes. In fact, the remarkable variability of the 2n and NF values in different *Clarias* species indicates that distinct chromosomal rearrangements occur during the evolution of their karyotypes. Karyotypes and other chromosomal characteristics of the three species in this study confirmed the patterns found for other *Clarias* species*,* except *C. batrachu*s (2n = 104). However, *Clarias* and related cytogenetic parameters warrant deeper discussion.

The walking catfish *C. batrachus* is native to Southeast Asia, but has been introduced outside its native range, where it is considered an invasive species responsible for invading aquaculture farms and preying on fish stocks [10, 11]. Indeed, C. *batrachus* has type locality in Java [12], while the populations from Indochina either represent introduced stocks or belong to other *Clarias* species, as a number of different species have been recently identified both in Indochina and in the Sunda islands [13]. Accordingly, cytogenetic data also point to very distinct karyotypes for this species: i) 2n = 50 in Malaysia, ii) 2n = 50–54 in India, iii) 2n = 56 in China and 2n = 100–104 in Thailand, in addition to sex and B chromosomes in some of these populations (Table 1). This brief overview of species suggests that the *C. batrachus* in Thailand, with 2n = 104 and a karyotype dominated by one-armed chromosomes, may represent a different unnamed species.

The hypothetical 2n for Siluriformes, as described in studies of different species of this order, was proposed to be 2n = 56, with a karyotype composed mainly by m-sm chromosomes [14–16]. Chromosomal studies of species in the group Heteropneustidae, which is phylogenetically considered a sister-group to Clariidae [17], report that most of its members also have 2n = 56 [18]. *Clarias gariepinus*, with 2n = 56 chromosomes, as well as a higher number of two-armed chromosomes and few acrocentric chromosomes, retains the karyotype considered basal for Siluriformes. These data support the phylogeny proposed for this family based on mtDNA analysis [9], in which *C. gariepinus* is placed together with *C. anguillaris,* as both species contain 2n = 56 chromosomes [19]. The decrease in the 2n of other Clariidae species (such as *C. macrocephalus -* Fig. 2), suggests that chromosomal fusions also participated in the karyotypic differentiation in the family.

Phylogenetically the species *C. batrachus* shows a derived position in the family [9]. In the present study, this species presented an unusual 2n = 104 and a karyotype dominated by acrocentric chromosomes. The occurrence of such a high 2n could be indicative of a polyploidization event. However, when considering the large number of acrocentric chromosomes and their relatively small size compared to the chromosomes found in the other *Clarias* species, the present data suggest that multiple centric fissions are, in fact, the most plausible explanation for karyotype diversification in this species. A similar process culminating in an increased 2n number has also been reported for species of the genus *Potamorhina*. One species - *P. altamazonica -* with a 2n = 102, diverged from the most frequent 2n (54) found in other congeneric species through a process of multiple centric fissions. In that case, meiotic analysis showed only bivalents at metaphase I and confirmed a large scale occurrence of extensive chromosomal fissions [20]. Additionally, extensive centric fission and heterochromatinization have been proposed in the karyotype diversification of the Alaska black fish (*Dallia pectoralis*) [21].

The chromosomal distribution of repetitive DNA elements revealed remarkable differences among the analyzed species. Both *C. gariepinus* and *C. macrocephalus* presented two 18S rDNA sites but were located in distinct chromosomal pairs, while four 5S rDNA sites were present in *C. gariepinus* and only two in *C. macrocephalus.* In addition, analysis of *C. batrachus* revealed six chromosomal pairs harbouring 18S rDNA sites and 27 chromosomal pairs harbouring 5S rDNA sites, including a synteny case. Though rDNAs are among the most conservative components of the eukaryotic genome, undergoing minimal changes over hundreds of millions of years, this conservatism appears to be a powerful source for genome instability [22]. Due to high similarity among clusters, chromosomes that carry extended rDNA arrays could be involved in heterologous synapses and recombination [23], providing variations of these sites inside the karyotypes.

Hypervariability in the number and location of rDNA loci, as presently reported in *C. batrachus*, has been previously described for several groups [24–26]. Variability in the number and position of rDNA sites suggested that chromosomal rearrangements played a role in the speciation of the plant *Sideritis dendrochahorra* [6]. This species possesses a large number of acrocentric chromosomes and multiple terminal 45S rDNA sites in most of its chromosomes. It has been suggested that in some groups, structural changes may be induced by selective pressures from ecological or environmental stresses [6, 27]. Among fishes, the spreading of rDNA has reportedly affected the recombination rates of two coexisting salmonid species, *Coregonus albula* and *C. fontanae*, leading to rapid genomic divergence and faster ecological speciation [28]. In some cases, transposable elements have been reported to play an important role in spreading rDNA sequences over the genome [24, 25]. Some classes of transposons appear to be able to “capture” entire genes and move them to other parts of the genome [29, 30]. Alternatively, several satellite DNA repeats may have originated from rDNA sequences and thus facilitate their dispersal into different genomic regions. For example in the fish *Hoplias malabaricus*, a highly amplified satellite repeat (5S*Hind*III-DNA) with sequence sharing similarity with 5S rDNA have been reported to exist in the centromeric region of several chromosomes [31, 32]. However, the reasons for the higher number of rDNA sites in *C. batrachus* still need to be clarified.

### Repetitive DNAs as a powerful tool for Clarias hybrids identification

Cultured catfishes are one of the most important commodities in Thailand’s domestic freshwater fish market, where *C. macrocephalus* is always preferred for consumption due to its better taste [33]. However, this species has a very slow growth rate and low disease resistance. At the end of 1980s, hybrid catfish production increased, with the most produced fish derived by crossing the Asian catfish (*C. macrocephalus*) and the African catfish (*C. gariepinus*) [34]; fast growth and high disease resistance made these species attractive to farmers [33, 35]. However, this hybrid form is currently abundant in all Thailand’s rivers, threatening wild catfish populations due to competition, predation, and genetic introgression [34, 36]. In fact, these hybrids are potentially able to interbreed with the parental species, which can lead to gene pool introgression, as has been reported for *C. macrocephalus* [33].

Karyotype similarity, such as that which exists between *C. gariepinus* and *C. macrocephalus*, enhances the success of hybridization and back cross of many species [37]. In nature, the occurrence of chromosome numbers around the modal values of the clariid species may suggest that speciation within this group is related to a high rate of hybridization that results from common spawning [19].

However, when hybrids and individuals from parental species have similar karyotype structures, the use of differential cytogenetic techniques is required to provide distinguishable chromosomal markers [38]. Indeed, several known hybrids can be precisely identified and clearly distinguished from their parental species using cytogenetic markers [38–40]. For example, conventional staining helped in the precise identification of the parental chromosomal types of the artificial hybrid resulted from the cross between *Colossoma macropomum* and *Piaractus brachypomus* [41]. In the present work, repetitive DNAs proved to be informative and allowed for precise characterization of the hybridization process. The hybrid *C. gariepinus* x *C. macrocephalus* had two 18S rDNA sites in non-homologous chromosomes in addition to three 5S rDNA sites; these were the exact intermediate numbers present in the parental species. In addition to the rDNA markers, microsatellites were highly useful for confirming the hybrid nature of the fish. These ubiquitous repeated sequences are present in all eukaryotic genomes, either in euchromatin or in heterochromatin, inside coding regions of structural genes or between other repetitive sequences [42]. In the current analyzed species, *C. gariepinus* presented faint hybridization signals of (CA)_15_ and (GA)_15_ at subtelomeric regions, while in *C. batrachus* and *C. macrocephalus* these sequences were highly accumulated in all chromosomes**.** Notably, the *C. gariepinus* x *C. macrocephalus* hybrid showed 27 chromosomes with the strong hybridization pattern, characteristic of *C. macrocephalus* chromosomes and the other 28 elements presenting a weak accumulation, found in *C. gariepinus.*

However, what are the genomic and ecological consequences of such hybrid unbalance? Interspecific hybrids not only led to diversification and speciation but also have important ecological consequences [43]. Some hybrids appear prevalent in nature, suggesting an evolutionary advantage for having different sets of chromosomes for adaptation and development [44]. It is known, for example, that hybridization can promote the activation of mobile elements and rapid genomic changes [45, 46]. Among fishes, interspecific hybrids between the red crucian carp (*Carassius auratus*) × common carp (*Cyprinus carpio*) showed faster genomic changes compared to the parental species, facilitated by intron gains and losses, homologous recombination and the formation of novel genes [47]. This ‘genomic shock’ has also been reported in many allopolyploid plants, translating as gene loss, chromosome mispairing, retrotransposon activation, altered methylation or rearrangements between parental genomes that could lead to novel gene sequences or differential homologous gene expression in hybrids throughout evolution [48].

## Conclusions

This study demonstrated that both conventional and molecular cytogenetics were useful tools in highlighting the remarkable chromosomal diversification that characterizes evolution in the genus *Clarias*. The distribution of repetitive DNA sequences on chromosomes identified: i) a high variability in the number and position of rDNA sites, ii) multiple chromosomal rearrangements, including an unusual number of centric fissions, iii) a high dispersion of ribosomal sites in *C. batrachus* and iv) the natural hybridization of *C. gariepinus* x *C. macrocephalus*. In the latter case, the hybrid genome was fully identified by the distinctive patterns of microsatellites sequences found in both parental species. Concerning conservation issues, these data stressed that successful natural hybridizations are not limited by low variations in diploid number or conspicuous divergences in microsatellites distribution among species.

## Methods

### Materials

**Fig. 4:**
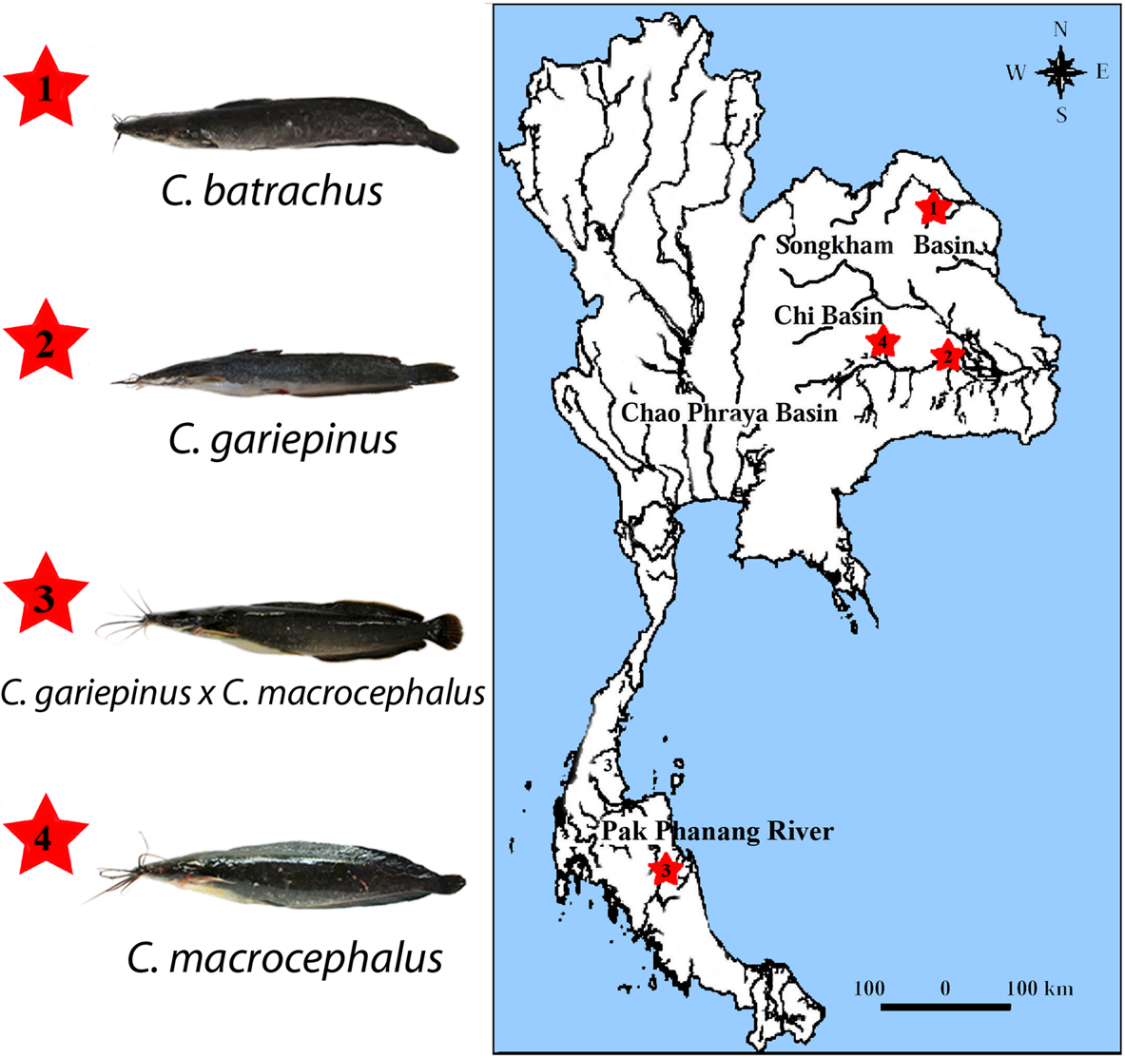
Collection sites of *Clarias* species from Thailand analyzed in the present study

Individuals of both sexes of three *Clarias* species collected from different river basins of Thailand were analyzed: *C. batrachus* (8 ♂ and 8 ♀), *C. gariepinus* (8 ♂ and 8♀) and *C. macrocephalus* (9 ♂ and 8 ♀); also collected were those from a probable natural hybrid between *C. gariepinus* x *C. microcephalus* (7 ♂ and 9 ♀) (Fig. 4). The specimens were caught using a hand-net, placed in sealed plastic bags containing oxygen and clean water, and transported to the laboratory. Experiments were performed in accordance with ethical protocols, and anaesthesia using clove oil was administered prior to sacrificing the animals to minimize suffering. The process was approved by the Ethics Committee of Khon Kaen University and by the RGJ committee under no. PHD/K0081/2556. Mitotic chromosomes were obtained from cell suspensions of the anterior kidney using the conventional air-drying method [49]. The specimens were deposited in the fish collection of the Cytogenetic Laboratory, Department of Biology Faculty of Science, Khon Kaen University.

### Chromosome probes and FISH experiments

Two tandem-arrayed DNA sequences isolated from the genome of an Erythrinidae species, *Hoplias malabaricus*, were used as probes. The first probe contained a 5S rDNA repeat copy and included 120 base pairs (bp) of the 5S rRNA transcribed gene and 200 bp of the non-transcribed spacer (NTS) sequence [31]. The second probe corresponded to a 1,400 bp segment of the 18S rRNA gene obtained from nuclear DNA using PCR [32]. The 5S and 18S rDNA probes were cloned into plasmid vectors and propagated in DH5α *Escherichia coli* competent cells (Invitrogen, San Diego, CA, USA).

The 18S and 5S rDNA probes were labelled with Spectrum Orange-dUTP and Spectrum Green-dUTP, respectively, using nick translation according to the manufacturer’s recommendations (Roche, Mannheim, Germany).

The microsatellites (CA)_15_ and (GA)_15_, were used as probes and were synthesized as described by Kubat et al. [50]. These sequences were directly labelled with Cy3 at the 5’ terminus during synthesis by Sigma (St. Louis, MO, USA).

Fluorescence *in situ* hybridization (FISH) was performed in highly stringent conditions on mitotic chromosome spreads [51]. Metaphase chromosome slides were incubated with RNAse (40 μg/ml) for 1.5 h at 37 °C. After denaturation of the chromosomal DNA in 70 % formamide/2× SSC, pH 7.0, at 70 °C for 4 min, the hybridization mixture (2.5 ng/μl probes, 2 μg/μl salmon sperm DNA, 50 % deionized formamide, 10 % dextran sulphate) was dropped on the slides. The hybridization was performed overnight at 37 °C in a moist chamber containing 2× SSC. The first post-hybridization wash was performed with 2× SSC for 5 min at 65 °C, and a final wash was performed at room temperature in 1× SSC for 5 min. Finally, the slides were counterstained with DAPI and mounted in an antifade solution (Vectashield from Vector Laboratories).

### Image processing

At least 30 metaphase spreads were analyzed to confirm the diploid chromosome number, karyotype structure and FISH results. Images were captured using an Olympus BX50 microscope (Olympus Corporation, Ishikawa, Japan) with CoolSNAP and Image Pro Plus 4.1 software (Media Cybernetics, Silver Spring, MD, USA). Chromosomes were classified as metacentric (m), submetacentric (sm), subtelocentric (st) or acrocentric (a) according to Levan et al. [52].

## Abbreviations

2n: diploid number
a: acrocentric chromosome
DAPI: 4′,6-diamidino-2-phenylindole
dUTP: 2′-Deoxyuridine-5′-Triphosphate
FISH: fluorescence *in situ* hybridization
FN: fundamental number
m: metacentric chromosome
NTS: Non-transcribed spacer
PCR: polymerase chain reaction
rDNA: ribosomal DNA
rRNA: ribosomal RNA
sm: submetacentric chromosome
st: subtelocentric chromosome
